# Targeting redox imbalance in neurodegeneration: characterizing the role of GLP-1 receptor agonists

**DOI:** 10.7150/thno.86831

**Published:** 2023-09-04

**Authors:** Puja Ghosh, Rosaria Anna Fontanella, Lucia Scisciola, Ada Pesapane, Fatemeh Taktaz, Martina Franzese, Armando Puocci, Antonio Ceriello, Francesco Prattichizzo, Maria Rosaria Rizzo, Giuseppe Paolisso, Michelangela Barbieri

**Affiliations:** 1 Department of Advanced Medical and Surgical Sciences, University of Campania Luigi Vanvitelli, Naples, Italy.; 2 IRCCS MultiMedica, Milan, Italy.; 3 UniCamillus, International Medical University, Rome Italy.

**Keywords:** ROS, Dementia, Diabetes, GLP-1RAs, Oxidative stress

## Abstract

Reactive oxygen species (ROS) have emerged as essential signaling molecules regulating cell survival, death, inflammation, differentiation, growth, and immune response. Environmental factors, genetic factors, or many pathological condition such as diabetes increase the level of ROS generation by elevating the production of advanced glycation end products, reducing free radical scavengers, increasing mitochondrial oxidative stress, and by interfering with DAG-PKC-NADPH oxidase and xanthine oxidase pathways. Oxidative stress, and therefore the accumulation of intracellular ROS, determines the deregulation of several proteins and caspases, damages DNA and RNA, and interferes with normal neuronal function. Furthermore, ROS play an essential role in the polymerization, phosphorylation, and aggregation of tau and amyloid-beta, key mediators of cognitive function decline. At the neuronal level, ROS interfere with the DNA methylation pattern and various apoptotic factors related to cell death, promoting neurodegeneration. Only few drugs are able to quench ROS production in neurons. The cross-linking pathways between diabetes and dementia suggest that antidiabetic medications can potentially treat dementia. Among antidiabetic drugs, glucagon-like peptide-1 receptor agonists (GLP-1RAs) have been found to reduce ROS generation and ameliorate mitochondrial function, protein aggregation, neuroinflammation, synaptic plasticity, learning, and memory. The incretin hormone glucagon-like peptide-1 (GLP-1) is produced by the enteroendocrine L cells in the distal intestine after food ingestion. Upon interacting with its receptor (GLP-1R), it regulates blood glucose levels by inducing insulin secretion, inhibiting glucagon production, and slowing gastric emptying. No study has evidenced a specific GLP-1RA pathway that quenches ROS production. Here we summarize the effects of GLP-1RAs against ROS overproduction and discuss the putative efficacy of Exendin-4, Lixisenatide, and Liraglutide in treating dementia by decreasing ROS.

## 1. Introduction

Environmental and genetic factors can promote neurodegeneration in the cerebral cortex, causing the development of dementia. Since the cerebral cortex controls personality, actions, memories, and thoughts, neurodegeneration in this area of the brain is associated with the development of cognitive impairment. Frontotemporal dementia, dementia with Lewy bodies, Parkinson's disease, and Alzheimer's disease (AD) are all conditions characterized by a progressive development and worsening of dementia 1. Estimates suggest that 60-70% of dementia cases are AD, while only 25% are vascular dementia 2. According to the World Health Organization (WHO), by 2030, the number of patients with dementia will increase up to 75 million, while the research output on dementia is expected to double between 2017 and 2025. Thus, the generic term dementia is a public health priority.

Although several factors cause the development of dementia, the redox balance plays a crucial role in the pathogenesis of neurodegeneration. The neurodegenerative process is accompanied by the inability of the cell to maintain the homeostasis of reactive oxygen species (ROS) and by an alteration of antioxidant molecules 3. ROS are free radicals and highly reactive ions such as singlet oxygen, hydrogen peroxide, hypochlorite ion, hydroxyl radical, and superoxide radical. Depending on their concentration, localization, and persistence, they intervene in multiple cellular processes and can promote either harmful or beneficial effect. Indeed, low levels of ROS are essential for the correct functioning of multiple signaling pathways, while high concentrations of ROS can promote cellular damage and other noxious effects. Therefore, both low and high levels of ROS are held to play a role in the etiopathogenesis of a plethora of diseases.

Several studies have highlighted that the increase of ROS leads to the dysregulation of various proteins, such as tau and beta-amyloid, as well as to immune activation, events typical of dementia 3. Numerous studies show that ROS also contributes to the oxidation of DNA, RNA, and lipid peroxidation, which causes neurodegeneration 4.

Diabetes mellitus is one of the main causes of increased oxidative stress in cells and tissues. In this regard, much scientific evidence demonstrates that hyperglycemia activates the electron transport chain, thus increasing ROS activity 5. Many research groups have demonstrated an increased risk for diabetic patients to develop cognitive decline, possibly due to the common pathophysiological mechanisms shared by diabetes and the development of dementia 6. In diabetic patients, the impaired insulin homeostasis in the brain, insulin resistance, and hyperinsulinemia contribute to amyloid proteolysis, the disruption of neurovascular coupling, and to impaired astrocyte trafficking, phenomena underlying the neurodegenerative process 7. Therefore, antidiabetic drugs controlling plasma glucose levels and insulin resistance could theoretically have the ability to reduce the risk of dementia. Preliminary proofs of efficacy for some antidiabetic medications in the treatment of dementia has already been provided, *e.g.* sodium-glucose cotransporter-2 inhibitor (SGLT-2i), glucagon-like peptide-1 receptor agonists (GLP-1RA), dipeptidyl peptidase inhibitors- 4 (DPP-4i), metformin, thiazolidinediones. GLP-1 (glucagon-like peptide 1) is a hormone produced by the small and large intestines, pancreatic alpha cells, and the central nervous system 8. Its release occurs after the meal when the L cells of the intestine perceive the presence of food in the gastrointestinal tract 9. When GLP-1 binds to its receptor (GLP-1R), it exerts various effects on different organs through multiple signaling pathways, including cyclic adenosine monophosphate (cAMP) protein kinase A (PKA), mitogen-activated protein kinase (MAPK), epidermal growth factor receptor (EGFR), Phosphoinositide 3-kinases (PI3K) and Akt (protein kinase B). GLP-1R is expressed in various tissues, including pancreatic islets, pancreatic ducts, kidneys, lungs, heart, skin, immune cells, central and peripheral nervous systems, hypothalamus, hippocampus, and cortex 10. The first oral formulation of the GLP-1RAs approved by the United States Food and Drug Administration was Rybelsus. Until then, only the injectable form was available. This is because GLP-1RAs are peptide or protein-based drugs, which can be easily degraded by the presence of proteolytic enzymes present in the gastrointestinal tract when consumed in tablet form 11.

Further, the GLP-1RAs can be characterized into i) long-acting agents, which include semaglutide, liraglutide, exenatide extended-release, and dulaglutide, and ii) Short-acting agents comprising lixisenatide and exenatide 12. Liraglutide has 90% peptide sequence homology with GLP-1, and its half-life can also be increased up to 13 hours by adding a fatty chain to it 13. That is why liraglutide is considered to be the full agonist of GLP-1. Researchers are trying different methods to develop an oral form of treatment. Bao and his team formulated a zein nano peptide hybrid loaded with exenatide and found promising results when administered to type 2 diabetic mice 14. In phase 2 clinical trial, oral formulation of semaglutide on type 2 diabetic patients showed better glycemic control compared to placebo 11. When loaded into chitosan-based nanoparticles and administered in mice, Liraglutide showed a significant increase in its bioavailability 15.

Diabetes mellitus and hyperglycemia promote broad damages in several organs. In particular, hyperglycemia induces the accumulation of intracellular ROS, causing oxidative stress in multiple tissues. This leads to a malfunction of the molecular mechanisms underlying cell survival, resulting in tissue damage. Multiple studies highlight promising results for GLP-1RAs in comparison to other antidiabetic drugs in the setting of attenuation of oxidative damage. However, the molecular mechanisms through which they exert their antioxidant action are only partially recognized.

## 2. ROS and their effects in the neurons

ROS are critical molecules in neuronal physiology and are involved in growth and development. In a balanced state, ROS play vital roles in several pathways, but when out of balance, they promote neurodegeneration. Even the cellular defense systems and the cellular metabolism determine an increase in ROS levels which, if not accurately balanced by the antioxidant systems, leads to oxidative stress. The latter is the cause of protein oxidation, DNA damage, and lipid peroxidation 16.

Ninenty percent of ROS are generated in the mitochondria, while only a minor part is generated via other cellular compartments, encompassing the endoplasmic reticulum, peroxisome, and the cytosol 17. ROS mainly originate in the mitochondria from a) increased electron transport system (ETS) activity and b) decreased conversion of superoxide ions and hydrogen peroxide to water 18. They can be produced by seven different enzymes (Complexes I and III, α-ketoglutarate dehydrogenase, aconitase, succinate dehydrogenase, α-glycerophosphate dehydrogenase, and dihydroorotate dehydrogenase), which reside in the inner membrane of the mitochondria and two others (monoamide oxidase and cytochrome b5 reductase) present in the outer membrane of the mitochondria 19. Furthermore, free radicals are also produced by enzymes such as nicotinamide adenine dinucleotide hydrogen phosphate (NADPH) oxidase, mitochondrial (m) adenosine triphosphate (ATP) sensitive K^+^channels (mKATP channels) and nicotinamide adenine dinucleotide phosphate oxidase 20. In physiological condition, ROS act as a critical regulator of neuronal plasticity and cognition 21. Moreover, neuronal differentiation and proliferation are upregulated by ROS levels 22. Further, the physiological concentration of ROS in neurons is involved in the development of hippocampal neuronal polarity 23.

Neurons are highly susceptible to slight variations in intracellular ROS levels as they have a high lipid content, a low amount of antioxidant enzymes, and a high oxygen intake 19. While physiological ROS have also emerged as positive regulators of the processes driving brain growth, higher ROS levels result in neuronal pathophysiology 24. Numerous scientific evidences highlight a strong correlation between oxidative stress, beta-amyloid aggregation, neuroinflammation, and neurodegeneration 25, 26. Neurodegeneration is held to be promoted by the polymerization, phosphorylation, and aggregation of tau and beta-amyloid, all molecular mechanisms exacerbated by neuronal oxidative stress. During the neurodegenerative process, neuronal cells in some brain areas undergo a block of autophagy, a key mechanism to eliminate abnormal protein aggregates including the amyloid plaque. Persistent inhibition of autophagy results in the accumulation of protein aggregates causing loss of neuron function and cell death 27. NADPH oxidase-dependent superoxide production leads to the loss of dopaminergic neurons in N27 rat and nigra neurons in adult mice and stimulates the formation of advanced oxidation protein products by triggering the production of excessive ROS 28, 29. Increased ROS in BV2 microglia cell lines induced p38 MAPK and JNK phosphorylation, subsequently triggering NF-kB inflammatory pathway and mitophagy in neurons 30. ROS upregulation has also been shown to activate NLRP3 inflammasome activation and cleavage of Gasdermin-d (GSDMD), causing pyroptosis. This was confirmed by cleavage of caspase-1, production of downstream mature interleukin (IL)-1β and IL-18, as well as rupture of the rapid cell membrane 29.

Oxidative stress can also alter the expression of the proteins of the Bcl2 family, which is involved in the anti-apoptotic process due to their ability to regulate mitochondrial permeability through modifications of the transition pore. In turn, neuronal apoptosis has been suggested as a relevant phenomenon in the development of cognitive impairment. ROS also plays an essential role in regulating epigenetic modifications by altering the expression of DNA methyltransferases, which catalyze DNA methylation. Similarly, ROS also led to the alteration of chromatin structure through changing histone acetylation, which led to the repression of different genes 31. As a result, these alterations promote the expression of several genes responsible for dementia-associated pathogenesis 20.

## 3. Role of ROS in diabetes-induced dementia

According to the International Diabetes Federation, 537 million people of age between 20 to 79 were diagnosed with diabetes in 2021, and the number might rise to 783 million by 2045 [Diabetes Facts and Figures, International Diabetes Federation 2021]. Many studies highlight the strong correlation between diabetes mellitus and cognitive decline, with a substantial increase in the risk of developing dementia in people with diabetes 6. A meta-analysis involving 1.4 million subjects evidenced a 40% increased risk of developing dementia in people with diabetes who have had hypoglycemic episodes 32. The risk of vascular dementia and AD is also elevated in people with pre-diabetes and further augmented by a long duration of diabetes 33. Indeed, a study on people aged between 70 and 89 suggests that both a long duration and an early onset of diabetes increase the predisposition to mild cognitive impairment 34. Such a relationship is strengthened by comorbidities such as obesity, depression, dyslipidemia, and hypertension, which could synergistically lead to cognitive dysfunction 35.

Recently, to understand the genetic link between type 2 diabetes and AD, a study was performed to recognize the overlapping gene signatures. SLC2A2 was identified as the crosstalk gene possibly linking these two diseases 36. Further, Hao and his colleagues carried out a genome-wide association study. They identified 395 shared single nucleotide polymorphisms between type 2 diabetes and AD, suggesting that the same pathogenic processes might underlie the onset of both 37. Interestingly, based on pathway analysis utilizing a non-negative matrix factorization approach, 241 candidate genes connected to both AD and type 2 diabetes were identified, and it was predicted that these genes contribute to the shared pathogenic characteristics of AD and type 2 diabetes 38. Due to these overlaps and correlations between type 2 diabetes and AD, the term "type 3 diabetes” was coined to refer to diabetes-induced AD 39.

Selected molecular markers are shared between diabetes and neurodegeneration, *e.g.* miRNAs. miRNAs are small non-coding RNAs that help regulate gene expression by silencing or activating mRNA transcripts 40. Many microRNAs are dysregulated in diabetic patients and might play a role in cerebrovascular complications. These neuropathological conditions stimulate the cognitive decline associated with dementia. Several studies have highlighted high plasma levels of microRNAs involved in the redox balance. Salama et al. demonstrated that higher expression of miR-132 was detected among patients with mild cognitive impairment 41. Furthermore, several studies have focused showed that cognitive decline and cerebrovascular disease are characterized by increased receptors for advanced glycation end product (RAGE) and by the reduction of brain-derived neurotrophic factor (BDNF) in conditions of hyperglycemia 42. Hyperglycemia per se is one of the most common causes of cellular damage from oxidative stress, with the consequent dysregulation of several molecular pathways involved in cognitive decline 43.

Diabetic patients are characterized by impaired glycolytic capacity, impaired acetyl-CoA activity, and impaired glucose metabolism. These changes lead to the accumulation of ROS in the mitochondria, causing their dysfunction 44. Mitochondrial ROS influence several physiological activities in the brain cells. Excessive mitochondrial ROS production can result in neuronal dysfunction and death, as discussed in the previous section 45, 46. As a result, ROS have been identified to be a crucial factor for type 2 diabetes-induced dementia due to their involvement in neuroinflammation, neurodegeneration, and neuronal death. Additionally, diabetes promotes a marked increase in protein or lipid glycation due to elevated circulating glucose levels, leading to the formation of advanced glycation end products (AGEs). AGEs suppress the cellular antioxidant system and promote ROS production 47. They were found to be involved in pathophysiological mechanisms leading to dementia and thus considered as a potential link between diabetes and neurodegeneration 48.

Hyperglcyemia fosters the activation of DAG-PKC-NADPH-oxidase (diacylglycerol-protein kinase C) pathways,promoting ROS generation. Hyperglycemia also activates phospholipases C and D, which aid in elevating DAG expression. This promotes the activation of PKC, which translocates the cytosolic elements of NADPH oxidase (Rac 1 and 2, low molecular weight G protein, p40phox, p67phox, and p47phox) to the plasma membrane. Here, these components combine with NOX2, which results in the production of ROS 49. Interestingly, NADPH-oxidase has been found to be upregulated in the frontal and temporal cortex, which suggests that elevated NOX-associated redox pathways may play an integral part in the progression of dementia 50.

**Figure 1:**
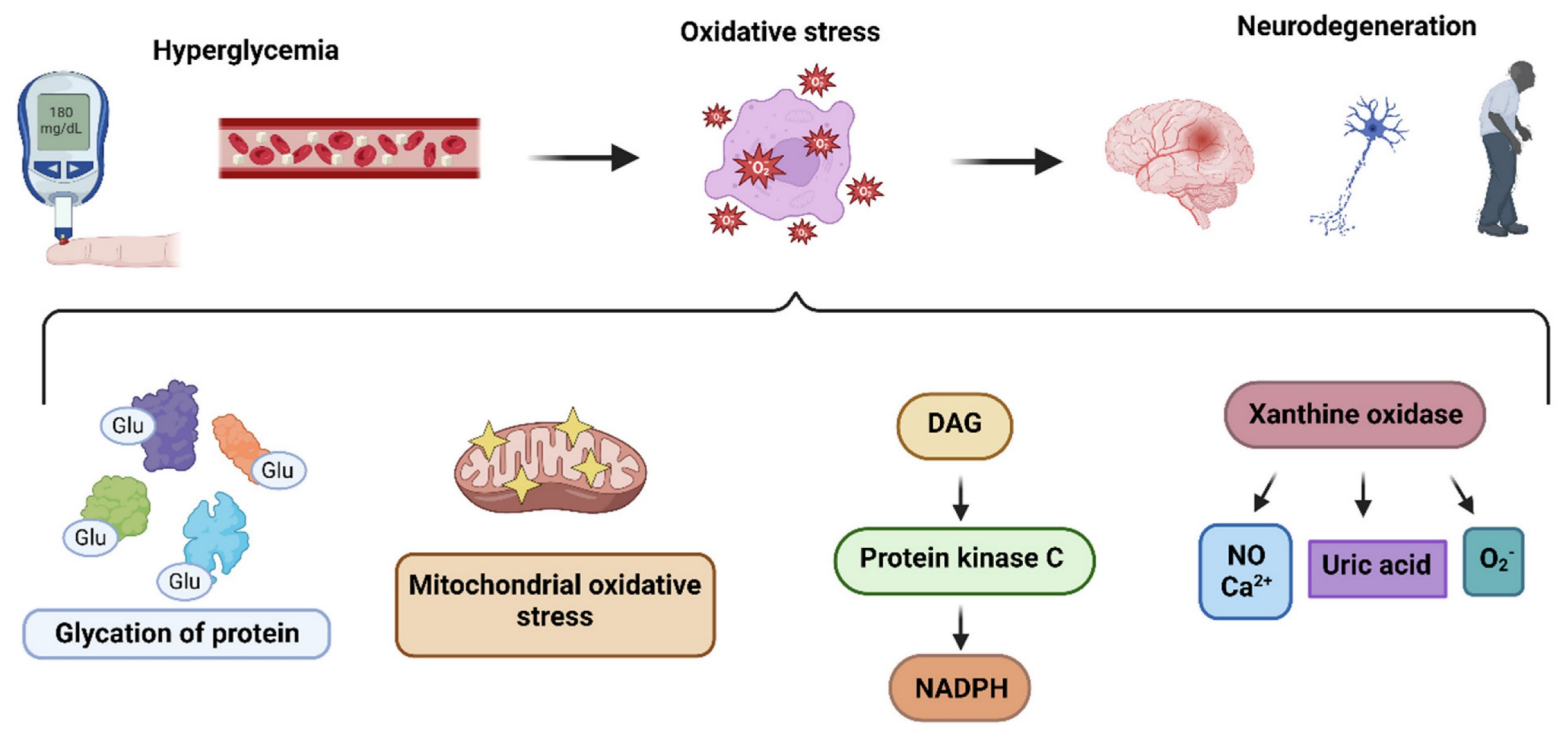
** Pathways leading to the production of hyperglycemia-induced ROS**: The hyperglycemia determines oxidative stress in nueronal cells, resulting in pathogenesis of neurodegeneration, through i) glycation of proteins ii) mitochondrial oxidative stress iii) DAG-PKC-NADPH pathway activation iv) xanthine oxidase upregulation.

Glucose, in the presence of hexokinase, a key glycolysis enzyme, is converted to glucose-6-P, which enters the pentose phosphate pathway and helps in purine synthesis. In the presence of purine nucleotide phosphorylase, purines are converted to hypoxanthine. In turn, xanthine oxidase converts hypoxanthine to xanthine, which eventually progresses to uric acid but also produces superoxide ions as byproducts. Hyperglycemia prompts the production of xanthine oxidase, which contributes to the generation of ROS through several pathways, including calcium signaling and nitric oxide 51 (**Figure 1**). Of note, Miric and colleagues found in their study that xanthine oxidase-induced ROS in diabetes patients contribute to the development of peripheral neuropathy, which is consistently associated with cognitive impairment 52, 53.

Hyperglycemiaalso interfers with the radical scavenging system. Different free radical scavenging mechanisms do exist such as zinc, copper, carotenoids, vitamin E, vitamin C, transferrin, albumin, lipoic acid, bilirubin, uric acid, and glutathione (GSH) 54. These molecules react with free radicals to form stable molecules, preventing chain reactions from destabilizing neighboring molecules. Interestingly, the postmortem frontal cortex of patients with different stages of AD demonstrated a considerable drop in antioxidants level 55. Selected evidences might also suggest that supplementing the diet with antioxidants might decrease the incidence of dementia 56.

## 4. Do GLP-1RAs prevent oxidative stress?

Recent studies highlighted many beneficial effects of GLP-1 in different tissues. It has been demonstrated that GLP-1 activates exchange proteins (Epac2), which stimulates insulin secretion and inhibits glucagon secretion by pancreatic cells 57. Indeed, GLP-1 is able to reduce the hepatic production of glucose, shows a protective effect at the cardiac and neuronal levels, reduces the oxidative stress of the vascular system, and induces the proliferation of pancreatic beta cells 58. Besides the well-known antihyperglycemic and protective effects in different cell types, GLP-1RAs regulate other cellular functions.

It is well known that diabetes mellitus, and therefore hyperglycemia, causes the accumulation of intracellular ROS in several organs and tissues. The ROS can affect physiological cellular functions, and all this causes diabetic complications. The accumulation of ROS appears to trigger cellular death and inflammatory molecules related to the pathogenesis of diabetes complications 49. Furthermore, ROS-induced inactivation of anti-atherosclerosis enzymes, AGE overproduction, upregulation of stress-sensitive signaling cascades, and impairment of insulin signaling pathways lead to the development of macrovascular (cardiomyopathy) and microvascular (neuropathy, nephropathy, retinopathy, atherosclerosis) diabetic complications 59, 60.

GLP-1-RAs were found to reduce ROS generation in several experimental models (**Table 1**). It has been shown that GLP-1 treatment could reduce ROS in diabetic rats as it can restore the expression of manganese superoxide dismutase (SOD) and catalase 61, 62. Because of this antioxidant property, GLP-1 is known to prevent other diabetic complications, including heart disease, neuropathies, peripheral vascular disease, and renal failure 63. *In vitro* analysis of HUVECs (human umbilical vein endothelial cells) revealed that GLP-1 could prevent endothelial dysfunction caused by type 2 diabetes mellitus by decreasing the level of ROS via the GLP-1R-ERK1/2 pathway 64. Furthermore, GLP-1RA treatment significantly reduced methylglyoxal-triggered ROS in cardiomyoblasts 65. In addition, increases in antioxidant proteins such as SOD, glutathione peroxidase, and catalase were observed in murine cardiac cells (H9c2) after treatment with Exenatide 66. Xiong et al. demonstrated that after GLP-1RA administration, a decrease in adiponectin expression was observed along with inhibition of ROS, resulting in improved vascular tone 67. Exendin-4 may mediate cardioprotection in neonatal rats by inhibiting oxidative stress via the Epac-dependent pathway 68. GLP-1RAs also reduce ROS levels in the diabetic rat aorta by inhibiting NOX4 and its subunits Ras-related C3 botulinum toxin substrate 1 (RAC-1) and p47phox. NOX4 is a major source of ROS in endothelial cells 61. Furthermore, GLP-1RAs can counteract diabetic nephropathy by suppressing ROS production and inhibiting AGE accumulation 69. Liljedahl and colleagues demonstrated the beneficial influence of GLP-1RA on oxidative stress in the kidney. They evaluated the renal tissue proteome of healthy mice and mice with streptozotocin-induced diabetes (STZ) receiving either vehicle or Liraglutide. After injection with STZ, there was a reduction of antioxidant enzymes such as catalase and glutathione peroxidase-3, critical enzymes for the response to oxidative stress. Liraglutide (GLP-1RA) restored antioxidant enzyme levels and improved renal histological lesions induced by diabetes 70.

## 5. Role of GLP-1RAs in controlling ROS in the neurons

### 5.1. Pathways by which GLP-1RAs regulate ROS

**Figure 2:**
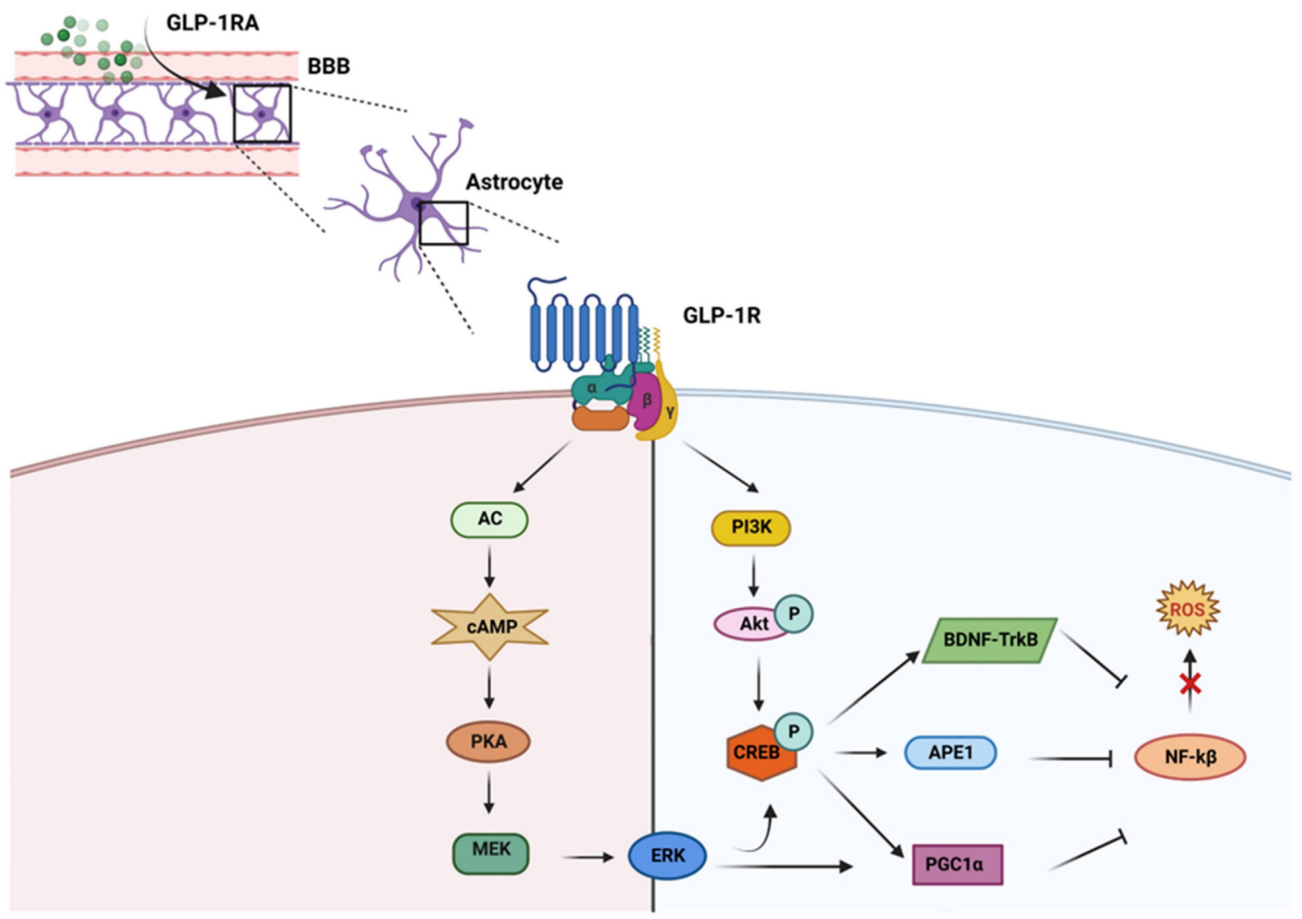
**GLP-1RAs regulate different pathways preventing ROS production:** GLP-1RA crosses blood brain barrier (BBB) and upon interacting with its receptor (GLP-1R) inhibits ROS upregulation mainly through two different metabolic pathways, i) AC-cAMP-PKA-MEK-ERK and ii) PI3K-Akt. Both these pathways activate CREB which in turn enhances the production of BDNF, APE1 and PGC1α. These molecules inhibit the upregulation of ROS by downregulating NF-kβ.

GLP-1 and its agonists can cross the blood-brain barrier (BBB) and affect central nervous system (CNS) functions 71. Larsson H. and colleagues highlighted that GLP-1RA signaling induces neurogenesis, reduces apoptosis, and protects neuronal function 72, 73. Besides the systemic level, GLP-1RAs are also able to reduce the production of ROS in neurons. ROS is essential in neurodegenerative diseases, especially AD pathogenesis and pathophysiology 74. In neurons, GLP-1RAs act on the reduction of oxidative stress through different metabolic pathways. Indeed, GLP-1RAs trigger the activation of i) AC-cAMP-PKA-MEK-ERK (adenosine cyclase-cyclic adenosine monophosphate-mitogenic activated protein kinase-extracellular signal-regulated kinase), and ii) PI3K-Akt (phosphatidylinositol 3 kinase-protein kinase B) when it binds to its receptor in neurons 75. These pathways activate the cAMP response element binding protein (CREB). CREB regulates the transcription of downstream genes BDNF, Apurinic endonuclease 1 (APE-1), and peroxisome proliferator-activated receptor 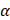 (PGC1α), which reduce ROS activity 76 (**Figure 2**). Furthermore, the multifunctional enzyme APE-1 has a nuclear localization signal and redox activity at its N-terminal end, thus decreasing nuclear ROS activity. Such later event interferes with the expression of several transcription factors such as signal transducer and activator of transcription 3 (STAT3), hypoxia-inducible factor 1-alpha (HIF-1α), AP-1, and nuclear factor-kappa B (NF-κβ), which in turn regulates inflammationand other cell signaling pathways 77.

GLP-1RAs help in increasing the production of antioxidant molecules, including ϒ-glutamate cysteine ligase catalytic subunit, peroxiredoxin sulfotransferase, UDP-glucuronyl transferase, glutathione S-transferase, glutamate cysteine ligase, glutaredoxin, glutathione reductase, heme oxygenase-1, NADP quinone oxidoreductase-1, sulfiredoxin, thioredoxin reductase, thioredoxin, glutathione peroxidase, catalase and SOD, through CREB-BDNF-TrkB signaling pathway 8. The activation of such antioxidant molecules blunts ROS 78. Since neurons have a high energy demand to support physiological neuronal activities, mitochondrial alteration occur during the neurodegenerative process with consequent impairment of neuronal functions 79. It is reported that GLP-1RAs can activate the PGC-1α signaling pathway, promote mitochondrial biogenesis, and reduce mitochondrial damage 80. Moreover, CREB also enhances the production of PGC1α, which reduces NF-kB and helps to decrease mitochondrial ROS production 81. Other researchers have also found that Exendin-4 significantly increases mitochondrial function, which is impaired by beta-amyloid accumulation 82.

GLP-1RA also interfere with activating a master regulator of cellular oxidative stress erythroid nuclear factor 2-related nuclear factor 2 (Nrf2) 8. Under normal conditions, Nrf2 is bound to Kelch-like ECH-associated protein 1 (Keap1) in the cytoplasm. Keap1 regulates the degradation of Nrf2 through the ubiquitination system. In oxidative stress conditions, the Nrf2-Keap1 complex breaks down, and Nrf2 translocates to the nucleus and induces the transcription of antioxidant genes 83. Interestingly, GLP-1RAs were discovered to enhance cellular antioxidant capacity through Nrf2 nuclear translocation via suppression of Keap1 and activation of the MAPKs, PKC and PI3K 84, 85.

### 5.2. Experimental evidence: GLP-1RAs controlling ROS

Recently, it has been demonstrated that GLP-1 shows positive neuro-regulation and protection effects in animal models 86 (**Table 2**). Liraglutide significantly decreased ROS overproduction in six months old 5x FAD mice and prevented other astrocyte mitochondrial dysfunctions 87. These results suggest that GLP-1R agonists can improve cognitive function by attenuating oxidative stress and mitochondrial dysfunction in the CNS. *In vitro* studies in SHSY5Y cells (human neuroblastoma cell line) suggest a marked reduction in oxidative stress and increased SOD levels when treated with Liraglutide 88. In the same cell line with silenced peptidyl-prolyl cis/trans isomerase (Pin1), another crucial ROS regulator, and treated with Liraglutide, there was an improvement in the insulin pathway and cell viability 89. Zheng and colleagues also demonstrated the neuroprotective effect of Liraglutide in SHSY5Y cells exposed to hydrogen peroxide (H_2_O_2_) 90. Further, Liraglutide in the AD mice model prevents beta-amyloid accumulation by rescuing oxidative stress 91. Liraglutide has also been shown to reduce p62 levels, an adaptor of lysosomal-mediated autophagy, in female mice with early AD-like pathology. P62 is important in oxidative stress and lysosomal-mediated autophagy 92. Other research showed that Liraglutide also downregulates 8-Hydroxy-2′-deoxyguanosine (8-OH-dG), a marker of oxidative DNA damage 93 and thiobarbituric acid reactive substances (TBARS), a significant marker for oxidative and nitrosative stress 94. In HT22 cells (immortalized mouse hippocampal cell line), GLP-1 reduces hydrogen peroxide generation and prevents neuronal death by reducing beta-amyloid aggregation, thapsigargin, tunicamycin, and L-glutamate 95. Incretins can also inhibit microglial death through the PKA pathway and upregulate several antioxidant enzymes' expression 96.

Several experiments in animal models highlighted the GLP-1RAs beneficial effect on ROS homeostasis. In neonatal Sprague-Dawley rats, the neuroprotective nature of Liraglutide was exerted by suppressing ROS production 97. In an AD-prone rat model, Exendin-4 administration was associated with remarkable cognitive performance and improved memory function 98. Excessive ROS production, induced by various stimuli, promotes the activation of NF-κB and increases proinflammatory cytokine expression 99. GLP-1 can reduce excessive ROS production through its anti-inflammatory properties. In neurons, microglia, and astrocytes, GLP-1 provides an anti-inflammatory effect by controlling receptor activation for advanced glycation end-products (RAGE), decreasing IL-1β (Interleukin-1 beta) and TNF-α (Tumor necrosis factor alpha) expression in the hippocampus, and inhibiting TLR4 (Toll-like receptor 4)/NF-kB signaling pathway 100. Moreover, treatement with Liraglutide is associated with a reduction of the proinflammatory cytokines IL-6 and IL-12p70 in the brain 101. In the Schwann cells isolated from a diabetic neuropathy rat model, treatment with Liraglutide decreased oxidative stress and attenuated inflammation 102. Taken together, these findings suggest a potential role for GLP-1RA in the treatment of neurological disorders such as dementia.

## 6. Role of GLP-1RAs in dementia

Dementia, especially AD, is a devastating neurodegenerative disease and a major cause of disability worldwide 103, 104. The available therapeutics for dementia can only manage some symptoms; therefore, researchers are focusing on developing a neuronal protective or regenerative drug that intercepts its pathogenesis. It has been demonstrated that, in several brain regions, like the hippocampus, nucleus accumbens, and striatum, the receptors of GLP-1 are expressed 105. GLP-1RAs improves memory, learning, and synaptic plasticity and inhibits neuroinflammation, protein aggregation, mitochondrial functions, and neuronal apoptosis 106. To deeply understand the role of GLP-1 in the brain, researchers generated knock-out mice for GLP-1 R, and they observed learning disabilities which got restored when the GLP-1 R gene was transferred to the hippocampal region 107.

Moreover, when a GLP-1RA is introduced into an AD rat model, a decrease in phosphorylated tauS396 and a reversal in memory impairment can be observed 108.

### 6.1. Evidence from human studies

Several studies were conducted to investigate whether GLP-1RAs prevent cognition impairment and functional deterioration in patients with dementia. Compared to the control group, the Exenatide group showed clinically significant improvements in cognitive decline 59. Life quality, daily activities, mobility, and non-motor symptoms were improved in patients treated with Liraglutide 109. In a clinical trial with 38 AD patients, treatment with Liraglutide was associated with a reduction in synaptic dysfunction and cognitive impairment 110. Additionally, data collected from double-blind randomized control trial patients receiving Dulaglutide showed a 14% reduction in cognitive impairment compared to placebo 111. Another study with 15000 participants also demonstrated the therapeutic benefit of GLP-1RAs in the decrease in the incidence of dementia 105. Gejl and colleagues highlighted that the patients treated with Liraglutide for six months showed remarkable improvement in cognition and a significant reduction in beta-amyloid load in the brain when examined through "Brief cognitive examination" and positron emission tomography (PET) imaging 110. In another clinical study, patients were enrolled after MINI International Neuropsychiatric Interview to understand their current mental problems and psychiatric history. Among them, patients with AD were treated with Liraglutide for 12 weeks. In order to investigate the neuroprotective effects of the drug, functional MRI (fMRI) of the brain was done both before and after the drug treatment. Results evidenced an increase in neuronal connectivity with the bilateral hippocampus in the Liraglutide-treated group compared to the placebo-receiving group 112.

Many clinical evidence highlighted the correlation between diabetes and neuropathology. Cheng et al. analyzed the fMRI of type 2 diabetes patients with cognitive decline. They showed remarkable restoration in the impaired cognitive domain after 16 weeks of Liraglutide treatment 113. It was also reported that when Liraglutide was given to 50 subjects with diabetes who were susceptible to dementia, very significant activation of the orbitofrontal cortex and dorsolateral prefrontal cortex brain regions was observed through functional near-infrared spectroscopy. The result of cognitive tests carried out on these patients after 12 weeks of treatment also showed better scores in attention and memory 114.

Moreover, another study conducted in people with diabetes and obesity evidenced that a treatment with with GLP-1RA for three monthsimproved the Montreal Cognitive Assessment (MoCA) score, which is a highly sensitive tool for the diagnosis of mild cognitive decline 115. Several studies demonstrated that insulin receptor desensitization (insulin resistance) is common in diabetes and dementia 116. Therefore, antidiabetic drugs can be employed to restore the activity of insulin receptors. In order to activate the desensitized insulin receptor in the brain, researchers focused on incretin mimetics which stimulates insulin through parallel signaling pathways. A clinical study with 38 AD patients receiving Liraglutide for six months showed increased brain glucose transfer capacity from 0.72 to 1.1 umol/g/min 117. Exenatide treatment on 15 male patients with type 2 diabetes resulted in an increase in glucose utilization in the brain which is required for glucose homeostasis regulation 118. Moreover, in a clinical study on 10 Caucasian males after GLP-1RAs treatment, the researchers used a PET scan and observed a decrease in brain glucose fluctuation dependent on plasma glucose modification 119.

Overall, these data suggest that GLP-1RAs have the potential to prevent neurodegeneration, restorate brain glucose signaling, and improve memory, learning, synaptic plasticity, and other cognitive functions in patients with dementia.

## 7. Conclusion

Diabetes activates several ROS-producing pathways in different tissues. In particular, in neurons redox imbalance results in neuroinflammation and neurodegeneration. Hyperglycemia-induced oxidative stress plays a significant role in the onset and progression of diabetic neuropathology. As a result, conditions characterized by cognitive impairment, such as Alzheimer's disease and vascular dementia, are more common in diabetic patients. Consequently, antidiabetic medications, particularly GLP-1RAs, can be repurposed to improve cognitive impairments in Alzheimer's patients. Available data support a beneficial effect of GLP-1RAs in inhibiting neuronal oxidative stress and other detrimental pathways for neurodegeneration, sustaining their potential role as candidates for treating diabetes-related dementia, possibly through their ability to counteract ROS imbalances in the brain.

## Figures and Tables

**Table 1:** Effect of GLP-1RAs on generating oxidative stress molecules in several tissues.

Author	Experimental model	Targeted tissue *in vivo*	Antidiabetic drug	Pathway	Oxidative molecule
Ji et al., 2022 120	C57BL/6 J mice AML12 cells (Hepatocytes)	Hepatic stellate cells	Liraglutide	Reduction of RAGE/NOX2	Hydrogen Peroxide
Cao et al., 2021 121	Sprague Dawley rats	Thoracic Aortas and Endothelial cells	Sitagliptin	Decrease in serum Malondialdehyde levels and increasing serum SOD	Reactive oxygen species
Chen et al., 2020 122	C57BL/6 mice	Cardiac fibroblasts	Liraglutide	Inhibition of Ang II-AT1R-ROS	Reactive oxygen species
Yang & Zhao, 2021 123	hRVECs cells (Human renital vascular endothelial cells)	-	Exenatide	Reduction of Sphingosine-1-phosphate receptor 2	Reactive oxygen species
Li et al., 2020 62	H9c2 cells (Cardiomyocytes)		Semaglutide	Activation of AMPK pathway, improves autophagy.	Reactive oxygen species
Wang et al., 2019 124	Diabetic Sprague-Dawley rat	Myocardial tissue	Exenatide	Inhibition of mammalian target of rapamycin complex 1/p70 ribosomal protein S6 kinase	Superoxide Radical
Ding et al., 2019 125	H9c2 cells (Cardiomyocytes)	-	Exenatide	Increase in the antioxidant enzymes manganese-dependent superoxide dismutase (MnSOD) and catalase.	Reactive oxygen species
Liljedahl et al., 2019 70	Male 129SV mice	Kidney	Liraglutide	Increase in protein glutathione peroxidase‐3 and catalase	Reactive oxygen species
Ke et al., 2017 126	HUVECs (Human umbilical vein endothelial cells)	-	Liraglutide + Metformin	Inhibition of PKC-NAD(P)H oxidase pathway	Reactive oxygen species
Chang et al., 2013 66	H9c2 cells (Cardiomyocytes)	-	Exenatide	Decrease in lactate dehydrogenase, creatine kinase, Malondialdehyde levels and increase in SOD, glutathione peroxidase & catalase	Hydrogen Peroxide

**Table 2:** Effect of GLP-1RAs on the generation of oxidative stress molecules in neurons.

Author	Experimental model	Antidiabetic drug	Pathway	Significance to human health	Oxidative molecule
Qi et al., 2022 88	SH-SY5Y cells (Neuroblastoma cells)	Liraglutide	Increase in SOD	Activation of energy metabolism and prevention of neuronal damage	Reactive oxygen species
Kornelius et al., 2022 102	RSC96 cells (Schwann cells)	Liraglutide	Inhibition of glucolipotoxicity	Attenuation of Schwann Cell's inflammation and dysfunction	Reactive oxygen species
Lambadiari et al., 2021 127	Type 2 diabetes clinical patients	Liraglutide + Empagliflozin	Induction of PKA & CREB, rise of glutathione	Increase of expression of neuroprotective proteins.	Reactive oxygen species
Xie et al., 2021 87	5x FAD mice	Liraglutide	Activation of the cAMP/PKA pathway	Enhancement astrocyte's neural support abilities	Reactive oxygen species
Duarte et al., 2020 91	3xTG-AD female mice	Liraglutide	Reduction of p62, 8-OH-dG, TBARS	Attenuation of beta amyloid accumulation and prevention of neuronal cells from oxidative and nitrosative stress	Reactive oxygen species
Bianchi et al., 2019 89	SH-SY5Y cells (Neuroblastoma cells)	Liraglutide	Induction of GSK3b/Akt	Reduction of neurotoxicity	Reactive oxygen species
Zheng et al., 2019 90	SH-SY5Y cells	Liraglutide	Influence Akt/GSK-3β	Prevention of AD induced neurodegeneration	Hydrogen peroxide
Spielman et al., 2017 96	THP-1 cells (human monocytes- microglia model)	Exendin	Activation of incretin receptors and PKA pathway	Upregulation of survival, and neurotrophins expression in microglia	Reactive oxygen species
Zhu et al., 2016 97	Neonatal Sprague-Dawley rats	Liraglutide	Activation of the PI3K/AKT and MAPK pathways	Prevention of neuronal apoptosis	Reactive oxygen species
Yoshino et al., 2015 95	HT22 cells (mouse hippocampal cell line)	GLP-1 (7-36)	Activation of Akt and ERK1/2 pathway	Protection of neurons from stressors	Hydrogen peroxide
An et al., 2015 80	PC12 cells (catecholamine cells)	Exendin-4	Reduction of andvanced glycation end products and tau hyperphosphorylation	Enhancement of mitochondrial biogenesis and prevent tau hyperphosphorylation	Reactive oxygen species
Chen et al., 2012 98	PC12 cells (catecholamine cells) and male Wistar rats	Exendin-4	Inhibiting high glucose-induced apoptosis	Protection of neurons against diabetes-related glucose metabolic dysfunction	Hydrogen peroxide

## Abbreviations

8-OH-dG: 8-Hydroxy-2′-deoxyguanosine
AC: adenosine cyclase
AD: Alzheimer's disease
ADAS: exec -Alzheimer's disease assessment scale-cognitive subscale and exclusive domain scores of the Neuropsychological test battery
ADP: adenosine diphosphate
AGEs: advanced glycation end products
Akt: (PKB) protein kinase B
APE-1: apurinic endonuclease 1
ATP: adenosine triphosphate
BBB: blood brain barrier
*Bcl2*: *B-cell lymphoma 2*
BDNF: brain-derived neurotrophic factor
cAMP: cyclic adenosine monophosphate
CNS: central nervous system
CREB: cAMP response element binding protein
DAG: diacylglycerol
DPP-4i: dipeptidyl peptidase inhibitors- 4
EGFR: epidermal growth factor receptor
Epac: exchange protein activated by cyclic-AMP
ERK: extracellular signal-regulated kinase
ETS: electron transport system
FAD: flavin adenine nucleotide
fMRI: functional Magnetic resonance imaging
GLP-1: glucagon-like peptide 1
GLP-1RAs: Glucagon-like peptide 1 receptor agonists
GSH: glutathione
H_2_O_2_: hydrogen peroxide
HIF-1α: hypoxia-inducible factor 1-alpha
HUVECs: human umbilical vein endothelial cells
IL: interleukin
Keap1: Kelch-like ECH-associated protein 1
MEK: mitogenic activated protein kinase
mKATP: mitochondrial adenosine triphosphate sensitive K^+^ channels
MnSOD: manganese-dependent superoxide dismutase
MoCA: Montreal Cognitive Assessment
MRI: magnetic resonance imaging
NADPH: nicotinamide adenine dinucleotide hydrogen phosphate
NF-κβ: nuclear factor kappa B
NOX: NADPH oxidase
Nrf2: erythroid nuclear factor 2-related nuclear factor 2
PET: positron emission tomography
PGC-1α: peroxisome proliferator-activated receptor
PI3K: phosphatidylinositol 3 kinase
Pin1: peptidyl-prolyl cis/trans isomerise
PKA: protein kinase A
PKC: protein kinase C
RAC-1: Ras-related C3 botulinum toxin substrate 1
RAGE: receptor for advanced glycation end-products
ROS: reactive oxygen species
SGLT-2i: sodium-glucose cotransporter-2 inhibitor
SOD: superoxide dismutase
STAT3: signal transducer and activator of transcription 3
STZ: streptozotocin
TBARS: thiobarbituric acid reactive substances
TLR4: toll-like receptor 4
TNF-α: tumor necrosis factor alpha
WHO: World Health Organization
